# How and Where Do We Ask Sensitive Questions: Self-reporting of STI-associated Symptoms Among the Iranian General Population

**DOI:** 10.15171/ijhpm.2018.18

**Published:** 2018-03-05

**Authors:** Maryam Nasirian, Samira Hosseini Hooshyar, Ali Akbar Haghdoost, Mohammad Karamouzian

**Affiliations:** ^1^Epidemiology and Biostatistics Department, Health School; and Infectious Diseases and Tropical Medicine Research Center, Isfahan University of Medical Sciences, Isfahan, Iran.; ^2^HIV/STI Surveillance Research Center, and WHO Collaborating Center for HIV Surveillance, Institute for Futures Studies in Health, Kerman University of Medical Sciences, Kerman, Iran.; ^3^Modeling in Health Research Center, Institute for Futures Studies in Health, Kerman University of Medical Sciences, Kerman, Iran.; ^4^School of Population and Public Health, Faculty of Medicine, University of British Columbia, Vancouver, BC, Canada.

**Keywords:** Sexually Transmitted Infection, Questionnaire, Self-report, Survey, Iran

## Abstract

**Background:** Reliable population-based data on sexually transmitted infections (STI) are limited in Iran and self-reporting remains the main source of indirect estimation of STI-associated symptoms in the country. However, where and how the questions are asked could influence the rate of self-reporting. In the present study, we aimed to assess what questionnaire delivery method (ie, face-to-face interview [FTFI], self-administered questionnaire [SAQ], or audio self-administered questionnaire [Audio-SAQ]) and setting (ie, street, household or hair salon) leads to more reliable estimates for the prevalence of self-reported STI-associated symptoms.

**Methods:** This cross-sectional study was conducted in winter 2014 on a gender-balanced (50.0% men) sample of 288 individuals aged 18–59 years old in Kerman, Iran. Respondents were recruited in (*a*) crowded public places and streets, (*b*) their households, and (*c*) hair salons. Data was collected on history of current and 6-month (ie, past 6 months) STI-associated symptoms. Three different methods including FTFI, SAQ and or Audio-SAQ were applied randomly in households and non-randomly in streets and hair salons to collect data among the respondents. Generalized estimating equation (GEE) was used to compare the settings and methods separately.

**Results:** A total of 2.8% of men and 9.4% of women self-reported at least one STI-associated symptom. Respondents were significantly more likely to report STI-associated symptoms when completing questionnaires on the street compared to their household (*P* = .0001). While women were less likely to report symptoms in FTFI compared to SAQ (*P* = .036), no significant differences were found between men’s responses across different methods (*P* = .064).

**Conclusion:** Further research is needed to evaluate the effect of different combinations of methods and settings to find the optimal way to collect data on STI-associated symptoms.

## Background

Collecting data on sexually transmitted infections (STI) is often laborious and prone to reporting biases.^1^ While data collection on STI prevalence could be carried out using various behavioural and biological data sources, self-reported data continues to be one of the main mechanisms for indirect estimation of STI across communities.^2,3^ Study setting (eg, household-based, street-based, or school-based) and method of questionnaire delivery (eg, interviewer- or self-administered) are two important factors that affect the reliability of sexual behaviours that are reported.^1,4^

A systematic review of similar studies in developing countries showed that different questionnaire delivery methods could notably influence the rate of self-reported information.^5^ The tendency to hide socially stigmatizing or ‘illegal’ behaviours (eg, drug use, extramarital sex) often lead to underreporting of the sensitive information in face-to-face interviews (FTFIs).^6^ In fact, a study suggested that respondents might be uncomfortable to answer some questions in front of an interviewer.^7^

Researchers have proposed several alternatives to FTFI to help address its weaknesses.^8,9^ Self-administered questionnaire (SAQ) invites respondents to complete an offline or online questionnaire on their own.^10^ SAQ has shown promise in eliminating some aspects of social desirability bias. However, this method is limited given its applicability to literate respondents only.^6,8^ Respondents may also misunderstand complicated questions in this method which could lead to response errors.^8,11^

Audio self-administered questionnaire (Audio-SAQ) is a revised form of SAQ in which the questions are pre-recorded and played for respondents over earphones, while they complete a paper copy of the questionnaire.^10^ Respondents listen to the recordings and respond according to the provided instructions.^6^ Elsewhere, in the context of STI-related topics, Audio-SAQ has been shown to create more privacy for respondents. For example, using Audio-SAQ in a study conducted in a STI clinic indicated higher reports of HIV-related risky behaviours compared to similar studies using other methods.^11^

Study settings is another important factor which can affect the reliability of self-reported data about risky sexual behaviours. A study in three culturally diverse cities in Iran (ie, Tehran, Urmia, and Kerman) compared people’s self-reported high-risk behaviours across three different settings (ie, household, telephone, and street) and found that individuals were less likely to report alcohol consumption or high risk sexual behaviours in household interviews compared to street-based interviews.^1^

In the conservative context of Iran and other similar socio-cultural settings, challenges around data collection facing STI researchers are even more pronounced. While it is essential to identify the most accurate questionnaire delivery method and setting for collecting data on STI-related topics, current data collection practices rely heavily on expert opinion and there is limited data-driven evidence on what works best among the Iranian general population.^2^ Therefore, we conducted this study to compare three questionnaire delivery methods (ie, FTFI, SAQ, and Audio-SAQ) as well as three data collection settings (ie, street, household, and hair salon) for collecting data on STI-associated symptoms among the general population of Iran. Findings of this study have important implications for Iranian STI researchers and health policy makers in designing their STI prevention studies.

## Methods

### Study Setting and Respondents

This cross-sectional study was conducted in the winter of 2014 to determine what questionnaire delivery method (ie, FTFI, SAQ or Audio-SAQ) and data collection setting (ie, street, household or hair salon) would result in the most reliable estimates for the prevalence of self-reported STI among the general population in Iran. This study was carried out in the city of Kerman which is located in the largest province of Iran. The population of 18–60-year-olds is estimated to be about 643 000 in Kerman. About 50% of population are male and 82% are literate. Moreover, 17% of men and 15% of women have some university education.^12^ Kerman was selected as the location of the study given its conservative setting and the logistics available to the research team that were located in Kerman. Women and men aged 18-59 years who lived in Kerman were eligible for the study but only literate respondents were eligible to complete self-administered questionnaires (SAQ and Audio-SAQ).

### Sampling

Assuming that 50% of the general Iranian population aged 18–60 years have ever had an STI-associated symptom,^13^ and considering a 0.25% margin of error and 95% confidence coefficient, the design effect was estimated at 1.25. The final sample size was adjusted based on expert opinion (a committee consisting of a number of STI experts, researchers and epidemiologists) and was calculated as 288 individuals. The city was first divided into four municipal areas. Each area was considered as a strata, and then participants were recruited from (*a*) crowded public places and streets, (*b*) their households, and (*c*) hair salons during peak hours: 9 am to 12 pm and 3 to 7 pm in each area. Respondents were selected using quota sampling, taking into account gender groups (ie, men and women) and age groups (ie, 18-30 and over 30) proportionate to size. Three questionnaire delivery methods (ie, FTFI, SAQ or Audio-SAQ) were applied in three different data collection settings creating nine different study modes. An equal number of subjects (ie, 16 men and 16 women) were included in each of these nine modes. In each municipal area, we targeted recruiting 72 adults to the study including 8 individuals for each of the nine modes of questionnaire delivery. Table 1 shows the nine modes of questionnaire delivery that have been applied in our study.

**Table 1:** Nine Modes of Questionnaire Delivery Applied in the Study

**Method**	**Setting**												
	**Street**				**Household**				**Hair Salon**				**Total**
	**Men**		**Women**		**Men**		**Women**		**Men**		**Women**		
	**18-30**	**>30**	**18-30**	**>30**	**18-30**	**>30**	**18-30**	**>30**	**18-30**	**>30**	**18-30**	**>30**	
SAQ	8	8	8	8	8	8	8	8	8	8	8	8	96
Audio-SAQ	8	8	8	8	8	8	8	8	8	8	8	8	96
FFI	8	8	8	8	8	8	8	8	8	8	8	8	96
Total	96				96				96				288

Abbreviations: SAQ, self-administered questionnaire; Audio-SAQ, audio self-adminestreated questionnaire; FTFI, face-to-face interview.

### Data Collection

#### Data Collection Instruments

To collect data, two anonymous gender-specific questionnaires on history of current and 6-month (ie, past 6 months) STI-associated symptoms were applied. For women, data was collected on 12 symptoms: groin pain, dysuria, frequent urination, inguinal lump, rectal discharge, genital ulcer, pruritus, dyspareunia, postcoital pain, vaginal discharge, postcoital bleeding, and lower abdominal pain. The last three symptoms were replaced with urethral discharge and swollen/red scrotum among men so data was collected on 11 symptoms among men. Respondents were asked if they had experienced each symptom in turn. (Response options: yes, no, don’t know). They were asked about current symptoms and those experienced in the last 6 months. The questionnaire was designed by the research team and reviewed and corrected by a group of infectious disease specialists, obstetrics and gynaecologists, urologists, midwives and general practitioners during several sessions. Questionnaires were pilot-tested and standardized prior to study initiation. In total, 30 individuals including 15 men and 15 women from the general population participated in the pilot testing. We recruited people from all the age groups and from both high and low socioeconomic regions of the city.

The content validity of questionnaires was assessed by a panel of STI experts and clinicians. The Kuder Richardson reliability^12^ was found to be 0.75 for questions on STI-associated symptoms. No statistically significant correlation was found between most questions in the covariance matrix (*P *> .05).^3^ To implement Audio-SAQ, a gender-specific recording was prepared on an mp3 player for men and women that clearly read out every question and responses.

#### Questionnaire Delivery Methods

Eligible respondents were approached by our trained interviewers and briefed about the study aims and objectives of the study. In the FTFI method, respondents were questioned about their STI-associated symptoms and their answers were recorded on a paper questionnaire. In this method, interviewers communicated with respondents directly and, where necessary, provided them with explanations about the questions or responses. On average, respondents requested for more information once, during the interview. With FTFI, all interviews were gender matched.

In the SAQ method, a paper copy of the questionnaire was handed to the respondent who completed it without assistance. The respondents read the questions and wrote down their answers.

Finally, in the Audio-SAQ method, a paper copy of the questionnaire along with an mp3 player and a pair of earphones were handed to respondents who were told to wear the earphones, look at the questionnaire and push the play button on the mp3 player. A recording was played providing instructions on how to fill out the questionnaire. Next, questions were read to the respondents followed by a pause to provide time for the respondent to give their response. While the interviewer was present, he/she did not intervene unless a technical issue was faced with the device; therefore, respondents were not able to seek help from a third person with their responses. SAQ and Audio-SAQ respondents returned their completed questionnaires to the interviewer. Respondents were asked to fold their completed questionnaires so that only the blank page of the questionnaire could be seen before handing them over to the interviewer. The interviewer also immediately placed the questionnaire among other ones so that the respondents could see that their answers were not read on the spot.

#### Data Collection Settings

In selecting households, we were provided with a list of households in Kerman by the deputy of health at Kerman University of Medical Sciences, Kerman, Iran and then we used a systematic random sampling frame (every fourth household) in each municipal area.^14^ If the house appeared to be occupied but no one was home, interviewers returned at another time. If no one was home on the return visit, there was no eligible person in the house, or the eligible respondent refused to participate in the study, an adjacent house was approached. When visiting households, questionnaires were completed at the front door of the respondent’s house. However, if the respondent preferred to go inside the house, the interviewer would accompany them to their preferred location in the house.

In the street-based approach, we first outlined the crowded streets and public places in each of the 4 municipal areas. Since these places were not many in number, we tried to include all of them in the study. To recruit respondents on the streets, we approached people non-randomly throughout the week (including holidays) during rush hours between 9 am and 1 pm and between 4 pm and 8 am. Using data collected during the pilot study, where the estimated time for approaching a person was 5.6 minutes and for completing the questionnaire ranged from 13.2 minutes for SAQ and 13.9 minutes for FTFI to 14.8 minutes for Audio-SAQ, we were able to calculate that we needed to recruit a respondent every 20 minutes during each 4-hour interval. On average, comp­­­leting the questionnaire for each person in self-administered an­­d interviewer-administered methods took almost 4.2 minutes (standard deviation [SD] = 2.4) and in Audio-SAQ was about 7.3 minutes (SD = 3.3). Before conducting the study, selected streets were carefully observed and in each street, a relatively quiet place was chosen to complete the questionnaire- a place where people commuted less frequently and preferably had a sitting space (eg, bus station, benches in the corner of a park or in the sidewalks, or benches inside stores and malls). Following recruitment, eligible respondents were asked to go to these places and complete the questionnaire. Where a certain demographic was missing from a particular quota (eg, young women less than 30), we would only invite individuals who looked like they fit the missing quota (eg, younger looking women) to participate and then confirmed if they were eligible by asking them their age.

Lastly, to recruit people in hair salons, a non-random sample of men and women was selected. Hair salons were selected after discussing with a panel of STI experts in the Ministry of Health who believed: (*i*) a diverse demographic population of the society could be reached in hair salons, (*ii*) people tend to visit hair salons every month, and (*iii*) the waiting area of hair salons would provide sufficient time and space for the interview. A typical hair salon in Iran has a general waiting area with a few chairs where several customers may be waiting for their appointment. Women’s salons usually have one or two more separate rooms for providing specific services. The usual working hours are from 8:00 am to 20:00 pm and a wide range of services like haircuts, facial trims as well as colour and treatments services are offered to the customers.

To identify hair salons, a list of all hair salons was taken from the hair salons’ association. A simple random stratified sampling procedure was used to select hair salons in each of the four areas of the city (4 strata). In each area 6 salons (3 women and 3 men salons) were randomly selected making a total number of 24 salons. Following a convenience non-random sampling approach, individuals were first approached, then briefed about the study and finally those who fit the inclusion criteria and provided consent were included in the study. In hair salons, respondents completed their questionnaires in a quiet place or empty room, an arrangement made previously with the owner.

In selecting people in streets and hair salons, the interviewers approached a diverse sample of the pedestrians or the clients of hair salons and sought their interest about participating in the study after giving them a brief overview of the objectives of the research. It also should be mentioned that due to our limited resources, random sampling was not feasible in selecting people in streets and inside hair salons since we may have not been able to recruit enough participants given our limited resources.

All respondents were provided with an educational pamphlet about STI prevention and treatment.

### Data Analysis

Following data collection, questionnaires were double data entered into Stata software (version 11) and checked for data entry errors. Missing data was removed from the analysis given its low frequency (ie, <5%) and limited impact on the analysis. Descriptive statistical indicators including frequency, percentage, mean, and SD were used to describe the results. When analysing our data, we tried to be consistent with the ‘more is better’ assumption (ie, the more a symptom was reported corresponded with higher efficiency of the applied methods or settings).^15^ In order to examine the association between dependent variable (ie, self-reporting STI-associated symptom) and independent variables (ie, questionnaire delivery method and setting), we applied logistic regression odds ratio (OR). To address the probable correlation between an individual’s responses (eg, respondents who have experienced STI are likely to have reported multiple symptoms) and the clustering nature of responses for each respondent, the generalized estimating equation (GEE) method was applied.^16^

###  Ethical Consideration

Considering the conservative context of Iran and to ensure confidentiality of the individuals’ responses, only verbal consent was obtained. To obtain verbal informed consent, respondents were briefed about the study objectives and methods as well as the anonymous nature of the data collection procedures.

## Results

### Respondents’ Demographics

A total of 288 respondents aged 18–59 years old (50.0% men) were enrolled in the study. All respondents were urban residents and more than half were married or in a stable relationship (57.8%) at the time of the study. Table 2 presents the demographic characteristics of the respondents stratified by questionnaire delivery method. The mean age of respondents was 36.6 (standard deviation [SD]: 10.6 years). Overall, around 45% of respondents had some college/university education and 63.4% were married. There were no significant differences in demographic characteristics of respondents across all questionnaire delivery methods (*P* > .05). While there was a difference in respondents’ education (*P* = .005), this was caused by our study design, since our illiterate respondents were not included in SAQ and Audio-SAQ data collections.

**Table 2:** Demographic Characteristics of Eligible Respondents by Questionnaire Delivery Method

**Demographic Characteristics**	**FTFI (n = 95)** **No. (%)**	**SAQ (n = 98)** **No. (%)**	**Audio-SAQ (n = 96)** **No. (%)**	***P*** ** (χ2)**
age groups				
18-29	924 (24.49)	824 (25.00)	123 (24.21)	.922
≥30	374 (74.51)	472 (75.00)	372 (75.79)	
Gender				
Female	48 (50.53)	48 (48.97)	48 (50.00)	.976
Male	47 (49.47)	50 (51.02)	48 (50.00)	
Education				
Illiterate	4 (4.21)	0 (0.00)	0 (0.00)	.005
≤High school	18 (18.95)	6 (6.12)	20 (20.83)	
Completed high school	32 (33.68)	45 (45.92)	32 (33.33)	
College/university	41 (43.16)	47 (47.96)	44 (45.83)	
Marital status				
Single	18 (18.96)	18 (18.37)	23 (23.95)	.536
Married/stable relationship	72 (75.78)	79 (80.61)	70 (72.91)	
Divorced/separated	5 (5.26)	1 (1.02)	3 (3.12)	

Abbreviations: SAQ, self-administered questionnaire; Audio-SAQ, audio self-adminestreated questionnaire; FTFI, face-to-face interview.

### Participation and Withdrawal Rate

The overall participation rate was 78% which did not differ significantly among men and women (*P* = .074). Also, the overall refusal rate was 22% with 35 individuals in street-based settings, 14 at their households and 14 in hair salons who declined to take the questionnaires. “Not having time to participate” was reported as the main reason for non-attendance in the study and in each setting, a similar proportion of the respondents (ie, 3%; 1% in men and 5% in women) did not complete the study leaving half way through the questionnaire. The highest proportion of respondents who did not complete the study was among women completing the Audio-SAQ (7% vs. 1% in SAQ and vs. 1.5% in FTFI) (*P* > .05). The most important reason women gave for not completing Audio-SAQ was that it was time-consuming.

### Self-reporting Sexually Transmitted Infections-Associated Symptoms

A total of 2.8% of men and 9.4% of women self-reported at least one STI-associated symptom; a figure that varied by the time-point of having the infection, as well as study methods and settings (Figure). Overall, compared to street-based sampling approaches, respondents were significantly less likely to report STI-associated symptoms when completing questionnaires at their households (*P* = .0001). Women were less likely to report STI-associated symptoms in FTFI compared to SAQ (*P* = .036). No significant differences were found between men’s responses across different study methods (*P* = .064).

**Figure:**
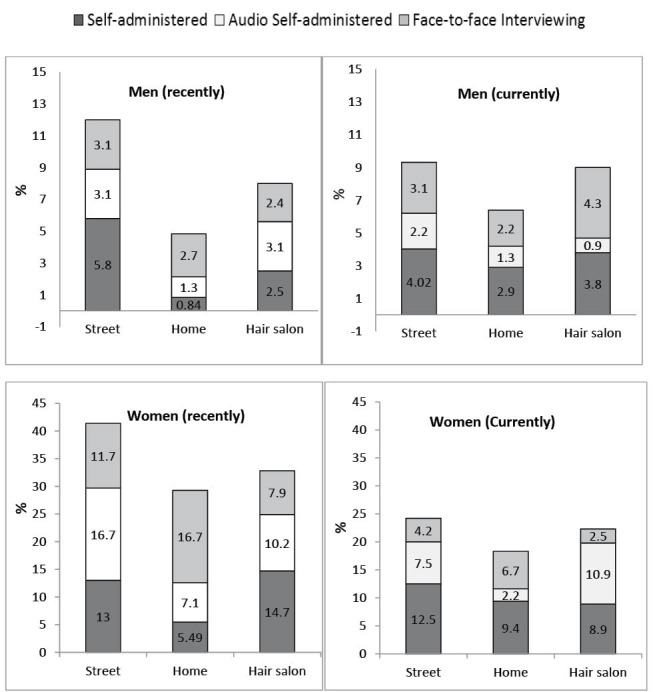
The Frequency of Responses to Sexually Transmitted Infections (STI)-Associated Symptoms, by the Time of Being Infected, Method and Setting of Study.

### The Association of Questionnaire Delivery Method with Self-reported STI-associated Symptoms

Details of the association of study methods with the current and 6-month (ie, past six months) self-reported STI-associated symptoms among men and women are presented in Table 3. Among women who underwent FTFI on the street, the odds of self-reporting STI-associated symptoms was 70% less than that of SAQ respondents (*P* = .002). Moreover, among those women who were approached at their household, the odds of self-reporting 6-month STI-associated symptoms in FTFI was 3.5 times more than in SAQ (*P* < .001). Lastly, among women who were recruited in hair salons, the odds of self-reporting current and 6-month STI-associated symptoms in FTFI was respectively 74% and 50% less than SAQ (*P* = .005 and *P* = .023). Conversely, our findings showed that the odds of self-reported STI-associated symptoms among men did not differ significantly across different study methods (*P* = .64).

**Table 3:** The Association of Questionnaire Delivery Method With the Individuals’ Responses About STI-Associated Symptoms

**Gender**	**Setting**	Method*	**OR (95% CI) of STI-Associated Symptoms**	
			**Current**	**6-Month**
Women	Street	SAQ	1	1
		Audio-SAQ	0.57 (0.31–1.05)	1.35 (0.81–2.23)
		FFI	0.30 (0.15–0.64)^a^	0.89 (0.52–1.35)
	Household	SAQ	1	1
		Audio-SAQ	0.58 (0.22–0.82)^a^	0.32 (0.63–2.77)
		FFI	0.63 (0.35–1.33)	3.44 (1.82–6.51)^a^
	Hair salon	SAQ	1	1
		Audio-SAQ	1.26 (0.69–2.31)	0.66 (0.38–1.14)
		FFI	0.26 (0.10–0.67)^a^	0.50 (0.28–0.91)^a^
Men	Street	SAQ	1	1
		Audio-SAQ	0.54 (0.18–1.65)	0.52 (0.20–1.34)
		FFI	0.77 (0.28–2.10)	0.52 (0.19–1.30)
	Household	SAQ	1	1
		Audio-SAQ	0.11 (0.45–1.75)	1.61 (0.26–9.67)
		FFI	0.75 (0.24–2.41)	3.25 (0.65–6.26)
	Hair salon	SAQ	1	1
		Audio-SAQ	0.05 (0.23–1.07)	1.25 (0.41–3.77)
		FFI	1.14 (0.44–2.92)	0.94 (0.28–3.14)

Abbreviations: SAQ, self-administrated questionnaire; Audio-SAQ, audio self-administrated questionnaire; FFI, face-to-face interview; STI, Sexually Transmitted Infections. ^a^ *P* value < .05.

### The Association of Data Collection Setting with Self-reported STI-associated Symptoms

Details of the association of data collection settings with current and 6-month self-reports of STI-associated symptoms among men and women are presented in Table 4. Among women who completed the SAQ, the odds of reporting current symptoms at hair salons were 25% lower than when they were approached at their households (*P* = .001). However, in case of completing SAQ for 6-month self-reported symptoms, those approached at hair salons and on the street were respectively 3 and 2.5 times more likely to report their symptoms than those approached at the household (*P* = .001 and *P* = .005).

**Table 4:** The Association of Data Collection Setting With the Individuals’ Responses About STI-Associated Symptoms

**Gender**	Method*	**Setting**	**OR (95% CI) of STI-Associated Symptoms**	
			**Current**	**6-Month**
Women	SAQ	Household	1	1
		Street	1.37 (0.78–2.43)	2.55 (1.32–4.93)^a^
		Hair Salon	0.75 (0.50–0.94)^a^	2.95 (1.54–5.69)^a^
	Audio-SAQ	Household	1	1
		Street	3.57 (1.30–9.78)^a^	2.61 (1.42–4.81)^a^
		Hair Salon	5.43 (2.06–14.30)^a^	1.48 (0.77–2.84)
	FTFI	Household	1	1
		Street	0.61 (0.27–1.37)	0.66 (0.39–1.11)
		Hair Salon	0.36 (0.14–0.39)^a^	0.43 (0.24–0.77)^a^
Men	SAQ	Household	1	1
		Street	7.27 (1.62–13.6)^a^	1.38 (1.10–3.77)^a^
		Hair Salon	1.29 (0.48–3.54)	3.05 (0.61–10.30)
	Audio-SAQ	Household	1	1
		Street	1.68 (0.39–7.12)	2.37 (0.61–9.31)^a^
		Hair Salon	0.66 (0.11–4.01)	2.37 (0.60–3.71)
	FTFI	Household	1	1
		Street	1.41 (0.44–4.52)	1.17 (0.39–3.54)
		Hair Salon	1.96 (0.65–5.95)	0.88 (0.27–2.95)

Abbreviations: SAQ, self-administrated questionnaire; Audio-SAQ, audio self-administrated questionnaire; FFI, face-to-face interview; STI, Sexually Transmitted Infections. ^a^ *P* value < .05.

Moreover, among women who completed using Audio-SAQ, the odds of current self-reported STI-associated symptoms on streets or at hair salons was 3.5 and 5 times higher than those approached at household (*P* = .013 and *P* = .001). Also women tended to report their 6-month symptoms 2.6 times more when completing Audio-SAQ on the street compared to in their households (*P* = .002). Lastly, among women who were recruited via FTFI at hair salons, the odds of self-reported STI-associated symptoms at the time of study and in the past 6 months were 64% and 57% less than these amounts when they were interviewed at their household (*P* = .04 and *P* = .004).

Conversely among men, the odds of current and 6-month self-reported symptoms was 1.4 to 7 times higher when completing the SAQ on the street compared to household (*P* = .001 and *P* = .025).

## Discussion

We examined three questionnaire delivery methods and three data collection settings to identify the most appropriate method and setting to collect data on STI-associated symptoms among the general population of Iran. We observed that both men and women were more likely to report STI-associated symptoms in the street-based approach. Moreover, while women were more likely to report STI-associated symptoms using the SAQ method, findings among men revealed no difference between SAQ and FTFI.

Our respondents’ higher likelihood to disclose their STI symptoms in the street-based approach could be due to the anonymous nature of data collection in this method which is in line with the previous body of literature suggesting that Iranians are more likely to disclose ‘sensitive’ information on the street.^1^ However, our findings using the street-based data collection should be interpreted with an eye to the setting’s proneness to reporting, selection, and social desirability biases.^3,17,18^

While questionnaire delivery methods did not affect self-reporting rates among men, women were more likely to report STI-associated symptoms when using SAQ compared to other questionnaire delivery methods. This may be due to the reassured confidentiality of responses in this method. Moreover, it is possible that reading the questions also enhances the concentration and accuracy of responses among individuals.^4,19^ Our results are comparable to a study among Japanese women showing that the reported cases of sexual abuse by an intimate partner in SAQ were higher compared to FTFI.^20^ However, findings of a similar study showed that in many cases, SAQ not only fails to improve the participants response rates, but may even cause bias.^8^ An existing body of literature also shows that Audio-SAQ could be more helpful than SAQ since it increases respondent’s comprehension of the question and provides a greater sense of privacy for respondents leading to more reliable responses.^21-23^ However, the majority of those who did not complete the study were women approached by the Audio-SAQ method stating that it was time-consuming. Since the questionnaire took less than 10 minutes, it seems that participants might have been likely to say ‘too much time” because of having to listen to the reading.

Although the questionnaire delivery method and data collection setting were investigated separately, it should be kept in mind that the selected setting for collecting information in different delivery methods may have different outcomes. For example, it seems that when women complete a SAQ in a hair salon or on the street, the self-reported rate is higher than when they are undertaking a FTFI in the same setting. But when data collection is taken in a household, self-reports are higher in FTFI compared to SAQ. This might be because women feel more relaxed or secure at their household and could answer questions with greater ease in their private environment. Also, despite its limitations in asking socially sensitive or censured behaviours, FTFI often provides the interviewer with a chance to present further details on each individual question and resolve misunderstandings.^9^ In a household, an interviewer might apply these advantages better than other settings since households usually offer a quieter, more peaceful and a much more private environment. All of these together may enhance the rate of self-reporting in household FTFI for women.

Women also tended to report STI-associated symptoms more frequently than men did. The possible explanation for such a difference might be that such symptoms are much less specific among women or women are more health conscious.^3^ Another reason is that women might really experience these symptoms more often since many of these symptoms are not caused solely by STIs. This finding is consistent with previous studies in Iran which suggest that women are likely to report their STI-associated symptoms more frequently than men.^3,13^

We acknowledge the limitations of our findings. Similar to other studies on collecting sensitive data, our self-reported data collected by convenience sampling are prone to recall, reporting, and social desirability biases. While some of these concerns could have been avoided through the use of cognitive interviewing which fix “the logic of the questionnaire,”^24^ our pilot testing of the questionnaire might have been helpful in addressing some of these concerns. Given our timeline and resources, we were not able to conduct cognitive interviewing or use approaches like audio computer-assisted survey instrument (ACASI) which has been proven to decrease reporting biases significantly,^5^ or factorial surveys and vignette studies that are less prone to social desirability bias^25-27^; however, it is recommended for future studies.

Our data were analysed based on “the more is better” assumption which has some limitations due to lack of having a suitable biomarker but has been applied in elsewhere and has been relatively admissible.^15^ Another possible bias might be that those participants who volunteered to take part in the study might have felt less threatened by the questions and therefore, might have been less likely to view the whole study as an invasion of privacy.

Furthermore, our findings may not be generalizable to the general population of Iran given the urban context of our data collection setting as well as the non-random nature of our street-based and hair salon-based samples. However, despite the arguable generalizability of our findings to other regions in Iran, we believe due to the similarity of culture and society across different contexts in other regions, our findings could provide implications and insights for future research in collecting sensitive data in Iran.

In conclusion, our findings suggest that street-based setting seems to result in more self-reports of STI-associated symptoms among both men and women in our setting. In addition, although different questionnaire delivery methods do not seem to make any difference in self-reporting rates among men, women tend to self-report more while taking the SAQ. However, reported rates of STI-associated symptoms also seem to vary within settings based on the method of delivery. Therefore, further investigations are needed to evaluate the impacts of different combinations of questionnaire delivery methods and data collection settings to find the best approach to gather data on the sensitive issues like STI in the conservative context of Iran.

## Acknowledgements

The authors would like to thank the participants for their time and the following organizations for supporting and funding the study: the Ministry of Health and Medical Education (MoHME), Tehran, Iran and the United Nations Population Fund (UNFPA), New York City, NY, USA.

## Ethical issues

The study was reviewed and approved by Kerman University of Medical Sciences, Kerman, Iran (code: IR.KMU.ECR.1394.171). Participants provided verbal consents and were assured about anonymity and confidentiality of the responses given in the questionnaires. They were also informed about the voluntary nature of their participation and that they could leave the study whenever they desired to. All data was reported as aggregate data and did not identify subjects as individuals.

## Competing interests

Authors declare that they have no competing interests.

## Authors’ contributions

Conception and design: MN and AAH; Acquisition of data: MN and AAH; Analysis and interpretation of data: MN, AAH, SH, MK; Drafting of the manuscript: SH, MK, MN; Critical revision of the manuscript for important intellectual content: SH, MK, MN; Statistical analysis: MN, MK; Obtaining funding: MN, AAH; Administrative, technical, or material support: MN, AAH; Supervision: AAH, MK.

## Authors’ affiliations

^1^Epidemiology and Biostatistics Department, Health School; and Infectious Diseases and Tropical Medicine Research Center, Isfahan University of Medical Sciences, Isfahan, Iran. ^2^HIV/STI Surveillance Research Center, and WHO Collaborating Center for HIV Surveillance, Institute for Futures Studies in Health, Kerman University of Medical Sciences, Kerman, Iran. ^3^Modeling in Health Research Center, Institute for Futures Studies in Health, Kerman University of Medical Sciences, Kerman, Iran. ^4^School of Population and Public Health, Faculty of Medicine, University of British Columbia, Vancouver, BC, Canada.

## Key messages

**Implications for policy makers**

- Understanding the impact of different questionnaire delivery methods and settings for self-reported data collection about sexually transmitted infections (STI)-associated symptoms could inform policies about optimal ways of creating reliable estimates of STI in the conservative context of Iran.
- Self-reporting rates were not affected by the questionnaire delivery method among men. However, women tended to report higher rates of STI-associated symptoms when using SAQ compared to other methods.
- Street-based sampling seems to be the most acceptable way of responding to sensitive STI-related surveys among both men and women in Iran.

**Implications for the public**

While the sensitivities around sexually transmitted infections (STI) are understandable within the Iranian context, the general population should be informed that implementing effective policies to curb STI will be only possible when accurate and reliable information is provided by people. Also, it would be beneficial for the public to know that the rates of STIs or similar infections they learn about in the media are highly dependent on how data is collected and should be interpreted with an eye to the sampling method’s limitations.
